# Visual Analytic Tools and Techniques in Population Health and Health Services Research: Protocol for a Scoping Review

**DOI:** 10.2196/14019

**Published:** 2019-10-28

**Authors:** Jawad Ahmed Chishtie, Jessica Babineau, Iwona Anna Bielska, Monica Cepoiu-Martin, Michael Irvine, Andriy Koval, Jean-Sebastien Marchand, Luke Turcotte, Tara Jeji, Susan Jaglal

**Affiliations:** 1 Rehabilitation Sciences Institute Faculty of Medicine University of Toronto Toronto, ON Canada; 2 Institute of Health Services and Policy Research Canadian Institutes of Health Research Ottawa, ON Canada; 3 Ontario Neurotrauma Foundation Toronto, ON Canada; 4 Library & Information Services University Health Network Toronto, ON Canada; 5 Department of Health Research Methods, Evidence, and Impact McMaster University Hamilton, ON Canada; 6 Centre for Health Economics and Policy Analysis McMaster University Hamilton, ON Canada; 7 Canadian Institutes of Health Research Ottawa, ON Canada; 8 University of Calgary Calgary, AB Canada; 9 Department of Mathematics University of British Columbia Vancouver, BC Canada; 10 British Columbia Centre for Disease Control Vancouver, BC Canada; 11 Department of Health Management and Informatics University of Central Florida Orlando, FL United States; 12 Universite de Sherbrooke Quebec, QC Canada; 13 School of Public Health and Health Systems Applied Health Sciences University of Waterloo Waterloo, ON Canada; 14 Canadian Institute for Health Information Ottawa, ON Canada; 15 Department of Physical Therapy University of Toronto Toronto, ON Canada

**Keywords:** data mining, data visualization, population health, public health, health services research, machine learning

## Abstract

**Background:**

Visual analytics (VA) promotes the understanding of data using visual, interactive techniques and using analytic and visual engines. The analytic engine includes machine learning and other automated techniques, whereas common visual outputs include flow maps and spatiotemporal hotspots for studying service gaps and disease distribution. The principal objective of this scoping review is to examine the state of science on VA and the various tools, strategies, and frameworks used in population health and health services research (HSR).

**Objective:**

The purpose of this scoping review is to develop an overarching global view of established techniques, frameworks, and methods of VA in population health and HSR. The main objectives are to explore, map, and synthesize the literature related to VA in its application to the two main focus areas of health care.

**Methods:**

We will use established scoping review methods to meet the study objective. As the use of the term visual analytics is inconsistent, one of the major challenges was operationalizing the concepts for developing the search strategy, based on the three main concepts of population health, HSR, and VA. We included peer reviewed and grey literature sources from 2005 till March 2019 in the search. Independent teams of researchers will screen the titles, abstracts and full text articles, whereas an independent researcher will arbiter conflicts. Data will be abstracted and presented using the Preferred Reporting Items for Systematic reviews and Meta-Analyses extension for Scoping Reviews checklist and explanation by two independent researchers.

**Results:**

As of late August 2019, the scoping review is in the full-text screening stage. Data synthesis will follow and the first results are expected to be submitted for publication in December 2019. In this protocol, the methods for undertaking this scoping review are detailed. We present how we operationalized the varied concepts of population health, health services, and VA. The main results of the scoping review will synthesize peer reviewed and grey literature sources on the main methods of VA in the interrelated fields of population health and health services research from January 2005 till March 2019.

**Conclusions:**

VA is being increasingly used and integrated with emerging technologies to support decision making using large data sets. This scoping review of the VA tools, strategies, and frameworks applied to population health and health services aims to increase awareness of this approach for uptake by decision makers working within and toward developing learning health systems globally.

**International Registered Report Identifier (IRRID):**

DERR1-10.2196/14019

## Introduction

In the first formal use of the term *visual analytics* (VA) in 2005, Thomas and Cook defined it as the “science of analytical reasoning facilitated by interactive visual interfaces” in their seminal book, Illuminating the Path [1,2]. VA techniques have proven helpful to professionals in gaining insights into the ever-expanding world of large complex datasets and unstructured big health care data [3,4].

### Beyond Traditional Statistical Analysis

Although VA moves from traditional to exploratory data analysis, it brings together fields of data processing, management, mining, analysis, information visualization, and human-computer interaction [5-7]. It takes the power of traditional statistical analysis further by promoting an understanding of data with effective visual interfaces [1,8]. Typically, a VA tool uses a dimensional database model, as opposed to a relational database, whereas the analyst uses visual tools to develop interactive graphic displays that can further *drill* down to help explore and present summarized data [3]. These techniques offer an edge over traditional statistical analysis, which is limited because of humans being vulnerable to information overload [8,9]. VA tools offer a combination of *analytics* and the *interactive visualization* engines [10]. The analytics engine component involves data storage, transformation, and analysis, whereas the visualization engine provides functionality toward data manipulation and display [10].

VA techniques in health care also make use of machine learning for mining and automated analysis [4,11]. As a multidisciplinary field, VA is more than data or information visualization; its approach combines analysis, visualization, and human cognition [7,12]. This enables deeper insights for planning interventions through analytical reasoning, taking advantage of human cognition in processing visual representations and human-information interaction [10]. Interactivity is an important characteristic of VA interfaces, providing decision makers with the ability to explore data from multiple aspects and allowing for meaningful and enhanced visual representations that can be used toward evidence-informed decision making [13].

### Visual Analytics in Health Care: Advantages and Applications

VA is an increasingly popular method for exploring, analyzing, and communicating results from complex big data in health [14]. Although it is increasingly applied in the clinical sciences, there is a lack of literature synthesizing VA methods, frameworks, and tools in population health and health services research (HSR) [15]. This is especially important with the rising demand from clinicians, administrators, patients, and policy makers for innovative means to answer complex questions [1,16]. Through this scoping review protocol, we present our methodology for the exploration of VA in the overlapping areas of population health and HSR to address this gap in the literature. The methodology presented will also be useful for future studies replicating similar concepts and for conducting reviews on related topics.

Given their high volume, variety, and velocity, much of public health data can be categorized as big data [10]. Ola and Sedig point to 4 major advantages of how VA can meet the needs of diverse users in public health, which can be extended to population health and HSR. These include overall flexibility to select the most suited visualization form, interaction control with data and information, nonlinear exploratory analysis, ability to provide tailored reports according to various audiences, and task adjustment for advanced and nonadvanced users alike [10].

One of the primary aims of population health and HSR is to better understand disease distribution and barriers to equitable care. Defined as “research with the goal of improving the efficiency and effectiveness of health professionals and the health care system [17],” HSR encompasses a large area of research. The concepts of HSR and population health are intertwined: first, in the purview of studying problems through an overarching population lens, and second, through a health systems lens. The *population health approach* brings together the two in their application toward health sector reform, allowing researchers to formulate proposals for the organization and delivery of health care systems [18,19].

The efficiency and effectiveness of VA in data analysis and communicating issues in health care are being increasingly utilized [8]. VA techniques can be applied to complex and multiple data sources, including administrative databases, text-based electronic medical records (EMRs), and multiple data sources. The value addition of VA can be best illustrated by a few examples: Alberta Health Services’ live dashboards on health service performance shows the service utilization by geography, type, and other variables [20]; population mobility from various sources for identifying pandemics through large interaction graphs and flow maps [15]; clustering of disease incidence and prevalence, broken down by seasonality and location [21]; detecting and promoting the understanding of spatiotemporal hotspots for emerging disease trends and associated factors using multisource complex spatiotemporal data [22]; complex gene-related data analysis to increase accuracy and avoid errors [23]; and exploring health events in geographic areas such as cities and towns to prevent hospital admissions [24].

### Gap in the Literature

Despite the increasing and varied use of VA techniques, the term *visual analytics* lacks an accepted definition in the field of health care and can imply different ideas and applications. We found the use of the term for dashboards in critical care to disease surveillance using spatiotemporal techniques. Considering the fast growth of VA to answer complex health care research questions, clarification and categorization of the term and its application are needed.

Our preliminary literature search revealed various methods, frameworks, and use cases developed primarily by computer scientists working in the fields of advanced data mining, machine learning, and analytics. Shneiderman et al’s seminal “overview first, zoom and filter, then details on demand” mantra lays down the most basic workflow tasks related to the type of data under study [25]. We similarly considered Chuang et al’s [26] development of tools for textual analysis and Munzer’s [27] 4-level nested model for the design and validation of visualization systems [28]. The field is fast developing, with multiple methods, frameworks, and tools that could have potential applications to health care data.

Recent reviews on VA in the clinical sciences show that the technique is being used for different conditions, specialties, populations, and levels of care [3,14,29]. In population health and HSR, VA techniques are being applied to complex questions, with varied applications ranging from hospital stay to decision support on pandemics [13,15,30]. While formulating our research objectives, we identified peer-reviewed and gray literature sources on VA methods, frameworks, and strategies in these fields, such as the use of multipanel graphs for epidemiologists [8], VA methods for studying electronic health records (EHRs) and anesthesiology [3], and spatiotemporal hotspots [22]. We also considered recent reviews related to VA. Wu et al’s review presents the various methods and approaches for evaluation of health visualizations and VA while identifying the best practices [31]. Similarly, Islam et al’s review summarizes data mining applications and theoretical perspectives in health care analytics [29].

### Novelty of the Scoping Review and Protocol

As the number of health-related scoping reviews steadily rise each year, so does the need for protocols that address specific methodological challenges [32,33]. This protocol is of interest because the subject is substantially complex to scope because of the following reasons: (1) the multidisciplinary and intersectional nature of VA, (2) the broad areas and overlapping subject matter that *population health* and *health systems research* cover, (3) the nondiscriminatory nature of the terms in searching for literature in databases, and (4) the necessity of formulating research solutions methodologically to address these major challenges. In this protocol, we outline how we overcame these challenges to design an innovative review that was feasible, while encompassing an important subject area that has not been covered in a review so far.

This protocol outlines the scoping review methodology related to examining the state of the science of VA in the areas of population health and HSR. We first define the concepts, objectives, and research questions, followed by the design and methods. We discuss the expected results and contributions from the scoping review. In addition, we outline the challenges and solution we developed, allowing for feasibility, while maintaining rigor in a subject area not covered so far. We also present how we operationalized the search strategy for the 3 major concepts—population health, HSR, and VAs—that was undertaken over a course of 3 months, with the help of a multidisciplinary team and a dedicated information specialist. The search strategy was externally peer reviewed. The protocol is innovative and would prove helpful to researchers working in related areas and other stakeholders as the methods are replicable for other sectors. Through this protocol, we further aim at a higher level of transparency in reporting methods, maximizing rigor through peer review, and avoiding duplication of efforts.

The proposed scoping review is novel in summarizing VA methods that have been applied to cases in population health and HSR, using structured or unstructured, complex big data from single or multiple source(s). Furthermore, we focus on the application, frameworks, and methods that involve actual, proposed, modeled, or simulated data with end products that can be valuable to population health and HSR practitioners. We expect a small degree of overlap with reviews on health informatics and data mining, given that the technique has only been recently taken up in health care sciences [29,31]. However, to the best of our knowledge, there is no synthesis of literature on the use and application of VA as an important and quickly developing method in the interrelated fields of population health and HSR.

### Objectives and Research Questions

The overall purpose of this scoping review is to develop an overarching global view of established techniques, frameworks, and methods of VA in population health and HSR, using any type of data. The main objectives are to explore, map, and synthesize the literature related to VA, including the use of the term *VA* and its application in population health and HSR [34]. We will specifically examine the extent and nature of the literature on use cases of VA tools, techniques, strategies, and frameworks.

On the basis of Joanna Briggs tools for conducting systematic scoping reviews, we defined the major constructs of the review under population, concept, and context [32]. As this is a review on the methods of data analysis, we do not constrain the review to a population. The concept includes VA in population health and HSR, distinguishing it from conventional data visualization techniques, at different levels of analysis, including health service access and utilization.

## Methods

### Guidance Frameworks

To guide the scoping review methodology, we will primarily use the guidance established by Arksey and O’Malley [35], with improvements suggested by Levac et al [36] and Peters et al [32], with recent adjustments made by Tricco et al [37]. Methodological steps included identifying the research question; identifying relevant studies; study selection; charting the data; and collating, summarizing, and reporting the results [35]. The latter 2 groups’ work helps with contextualizing these steps toward the specific review.

### Study Outcomes and Eligibility Criteria

We identified the research questions following extensive consultations with the protocol authors to clarify the concept and purpose of the review. We considered the major question of studying VA in population health and HSR with the varied terminology used in the literature. Delineating VA from concepts such as information data visualization, which may or may not be *interactive*, is considered a major challenge for the review. We sought to limit this challenge through developing a detailed a priori eligibility criteria for the literature, with the types of literature to be included. The eligibility criteria are presented in Textboxes 1 and 2. The criteria are not considered exhaustive and will be developed further during the review.

As recommended by Levac et al [36], we considered the intended outcome of the review, which was to develop an overarching global view of established techniques, frameworks, and methods of VA in population health and HSR. It was also necessary to define this concept in relation to the search strategy to make the review feasible in terms of its time and scope. We will report the results based on the Preferred Reporting Items for Systematic reviews and Meta-Analyses extension for Scoping Reviews checklist and explanation [37].

**Inclusion criteria for literature.**

Inclusion criteria

1. Peer reviewed or conference papers.
2. Articles on population-level metrics: access, utilization, disease/condition distribution, and social/multiple determinants of health.
3. Articles that have interactive or exploratory techniques for spatial, temporal, spatiotemporal, and geospatial visualizations.
4. Articles on electronic medical and health records but with a clear population level or health services research component.
5. Articles with machine learning, natural language processing, automated analysis, data mining techniques, interactive tools, and iterative analysis.

**Exclusion criteria for literature.**

Exclusion criteria

1. Editorials, projects, or reports.
2. Studies conducted in clinical settings.
3. Articles for individual condition(s) from a single hospital/unit, such as intensive care, surgery, and anesthesia without a population component.
4. Articles on device or sensor data without a population component.
5. Studies that include static data/information visualization/techniques, including simple bar graphs.
6. Studies that do not include an analytic component or do not use big data sources.

### Identifying Relevant Literature

#### Operationalizing Concepts of Population Health and Health Services Research

One of the major challenges for this review was operationalizing the concepts for developing the search strategy. To aid with this, the scoping review team includes an information specialist (JB). The search strategy is based on 3 main concepts: population health, HSR, and VA. Population health has only recently been added as a Medical Subject Headings term in 2018 in MEDLINE [38], whereas *health services research* and *visual analytics* are nonspecific search terms. We detail the steps taken for operationalizing these concepts below.

We studied recent systematic and scoping reviews for the search strategies employed for operationalizing the population health, health services, and VA concepts. Table 1 describes the concepts, sources, and search terms extracted. For population and public health, experts have attempted to develop a common language related to what both areas and terms encompass [39]. Alpi et al described the challenges in searching the literature because of the broad nature and often interchangeable and overlapping use of both terms [40]. Researchers take varied approaches toward strengthening their search strategies. In a review of the methods and application of EHRs to population health, search concepts ranged from infectious disease to social epidemiology [41]. Two reviews strengthened the searches using a broad search strategy and filtering studies based on objectives and eligibility criteria during the screening stages [42,43]. Searches were also complemented with citation and cross-reference methods for identifying the relevant literature [43].

For operationalizing the population health concept, we gathered related terms used by the national public health language created by the National Institute for Health and Care Excellence, UK version 1.2 [44]. We then identified the relevant search terms from detailed database trees. We also compared search terms from these sources and 5 recent reviews in population health [19,41,45-47], for example, Fone et al presented a detailed search strategy for population health [46], which we adapted for our use.

As for HSR, we searched recent reviews for operationalizing the concept in combination with the filters developed by the National Library of Medicine [48]. We identified and used 4 reviews [18,49-51] to translate the concept to the search strategy (Table 1).

**Table 1:** Operationalizing concepts and search terms from reviews on population health, health services research, and visual analytics.

Concepts and terms	Reviews mentioning the search strategy and terms
Population health; population health; public health; surveillance: sentinel, biosurveillance, and population health; spatial epidemiology; spatiotemporal; precision: public health, population health, and surveillance; population health outcomes	Etches (2006) [45]; Cohen (2004) [19]; Fone (2003) [46]; Singh (2014) [47]
Demographic; preventive health; population health	The National Institute for Health and Care Excellence (2007) [44]
Geographic information systems; environmental epidemiology; social epidemiology; health determinants	Casey et al (2016) [41]
Health services research; health services research; health care/data; equity and inequity; process; outcome; appropriateness; access to care; utilization/health care utilization/health service utilization; quality/quality improvement; cost; outcome disparities; system interventions; planning; geospatial; decision support; evaluation; monitoring; financing; health service delivery	Health services research filters developed by the US National Library of Medicine [44]; Canadian Institutes of Health Research (2018) [17]; Thompson et al (2016) [49]: no search strategy, uses CIHR 2018 definition; Gulley et al (2018) [50]; Rowland et al (2014) [51]; Harris et al (2012) [18]
Visual analytics; visual analy* ; data visualization; interactive; automated analysis; dashboard; information visualization; data exploration; interactive data exploration; mathematical simulation/modeling with visualization/dashboard; big data analytics; predictive analytics; descriptive analytics; prescriptive analytics; big data	Reviews: Islam et al (2018) [29]; West et al (2014) [52]; Simpao et al (2014) [3]; Wu et al (2019) [31] and seminal articles: Basole et al (2015) [53]; Basole et al (2015) [54]; Caban and Gotz (2015) [16]; Falster et al (2014) [24]; Hosseinpoor et al (2018) [55]; Huang et al (2015) [56]; Martinez et al (2016) [57]; Ratwani and Fong (2015) [58]; Simpao et al (2015) [14]

#### Operationalizing Visual Analytics

VA is a specific term denoting specific approaches and techniques [1]. However, the term is vague, and alternate terms such as *data and information visualization* are used in the literature. We identified and used 4 major recent reviews related to the subject [3,29,31,52], along with 9 seminal papers [14,16,24,53-58], to identify the search terms (Table 1).

#### Defining Interactivity in Visual Analytics

Interactivity is usually stated to be one of the recent hallmarks of VA applications, owing to the manipulation of visual interfaces afforded by computing power [10]. We borrow from Ola and Sedig’s and Pike et al’s work to define *interactivity* as the ability to reflect changes in the visual representation of data based on one or more indicators available on the analytic interface to the user [10,59]. Pike et al categorized interaction elements into 2 main types: (1) *lower level* aimed at change of the visual representation to study patterns, relationships, and (2) *higher level* offers understanding of the intent of interaction itself toward knowledge discovery [59]. For selecting appropriate literature as part of this scoping review, we mainly focus on the *lower level* interaction that would allow tasks such as filtering; determining ranges; and finding anomalies, clusters, and the like by providing menus, dropdowns, and other options on the visualization interface. We expect to increase the accuracy for selecting VA literature, while having minimal overlap with other noninteractive visualizations that typically would not fall under VA. In addition, we will focus on VA literature that uses advanced techniques within the analytic engine, such as machine learning and natural language processing.

#### Final List of Search Terms

For developing the final list of search terms, the process for each concept was not necessarily linear. We constantly compared the list of terms from each step within each concept with detailed database trees to check that we included relevant concept components, while avoiding duplication of results. Following this methodology, we were able to control the *noise* in constructing the search strategy, making it manageable and feasible.

#### Search Strategy: Peer-Reviewed Sources

We have limited this review to formal VA methods, applications, and tools that have been either published as peer-reviewed literature or as full conference papers. Using the 3 main operationalized search concepts, the information specialist (JB) developed a search strategy in MEDLINE (Multimedia Appendix 1). We validated the search strategy by ensuring that it captured the key seminal studies about VA in population health and health care, in general, to ensure that the subject literature was included as broadly as possible. We limited the search to English language articles from 2005 onward to coincide with the formal use of the term *visual analytics* by Thomas and Cook in their seminal book, Illuminating the Path, in 2005 [1,2].

For fine-tuning and improving the search strategy for peer-reviewed articles in health-related databases, the strategy was peer reviewed by another information specialist at the University of Calgary, using the latest Peer Review of Electronic Search Strategies: 2015 Guideline Statement [60]. After incorporating the suggested revisions, the MEDLINE search yielded 4563 articles and included all 12 seminal studies. The MEDLINE search strategy will be adapted to EMBASE and other databases. Databases that are not primarily health related, such as from geography, mathematics, computer science, or engineering disciplines, will not be searched.

#### Capturing Gray Literature and Complementary Searching

During our initial searches, we realized that VA methods and representations such as dashboards are presented at conferences in real time, whereas the proceedings include full papers. Given VA’s fast development, this was considered a rich resource that differs from peer-reviewed literature.

We will capture the gray literature through translating the MEDLINE search to Web of Science, Compendex, and Inspect to identify full conference papers. Conference abstracts were excluded from the search for reasons of clarity and completeness of information. An abbreviated search will also be conducted in IEEE Xplore, a subject-specific database. In addition, we will complement our strategy with the searching of reference lists within peer-reviewed and gray literature sources and hand searching subject-specific journals and conference proceedings. These include *Applied Clinical Informatics*, *Visual Analytics in Healthcare*, *IEEE Transactions on Information Technology in Biomedicine*, *Journal of Medical Internet Research*, *Journal of Medical Systems*, *Journal of the American Medical Informatics Association*, *Health Affairs*, *Journal of Biomedical Informatics*, *Healthcare Informatics Research*, and *PLoS ONE*. Both the IEEE Xplore search and the list of journals are based on Islam et al’s review on data mining in health care [29] and a Web search by the authors. A Google Scholar and Google Web search engine review will also be conducted, limited to the first 100 results on both platforms.

We will not include dissertations, theses, and book chapters in the review. Furthermore, we will not search for data visualization websites, as it was deemed impossible to gather this huge body of data, adjudicate on the methods and results, and synthesize findings. In addition, frequent content and hyperlink changes would render these sources unusable to future readers in a short time. All citations retrieved will be amalgamated and managed using Clarivate Analytic’s Endnote citation management software [61].

### Study Selection

A priori selection criteria have been developed, which will be modified during the study selection process, as required. Following the methodology suggested by Levac et al, 2 reviewers will independently review the titles and abstracts to categorize whether the piece of literature is eligible for full review [36]. We expect at least 8000 articles, each of which will be randomly assigned to 2 reviewers. Studies that do not fall in either category or represent conflicts between the reviewers will be resolved by an independent referee. We will retrieve full-text results for the included studies and any unresolved studies for inclusion. In addition, 2 reviewers will screen the full-text results independently for inclusion in the next stage of the review. An expert third party will adjudicate in case of unresolved decisions for inclusion of studies at any stage. We will use the DistillerSR Web platform for efficiently managing the title and abstract review, full-text screening, and abstraction of data [62].

We will include published and in-press peer-reviewed articles, conference papers, and relevant gray literature sources that include quantitative, qualitative, and mixed method studies, tools, frameworks, and methods of the use of VA. All studies that mention *VA* as a method in population health and HSR, and at any level of both of the latter concepts, will be included, for example, VA methods for assessing an emergency room population over time will be included. Single disease visualizations at any facility or geographic level will not be included in the scoping review if these are meant to be used toward clinical decision making. However, any studies dealing with population metrics and health services indicators will be included. Furthermore, we will not include methods that may have an application to health care but were not applied to an actual or hypothetical health care research data. This is important as we limit ourselves to the application of VA to health care. We also include studies on EMR/EHR data if the research question or application is in population health or health services.

VA applications also borrow from and overlap with machine learning and natural language processing and can involve complex datasets, unstructured text data such as from EMR sources, and linked analysis. We will include and focus on articles that include any type of mining, querying, and analysis technique that includes VA application to HSR and population health. However, we will not include articles related to data preparation/harmonization, user experience and preference, and human-information and human-computer interaction. The eligibility criteria are given in Textboxes 1 and 2.

### Data Extraction

A data abstraction form will be developed and pilot tested by 2 teams composed of 2 researchers each, all working independently of each other. The data form will be tested on 5 to 7 articles for consistency and comprehensiveness for capturing relevant data. Changes will be made in a team meeting to discuss and compare the pilot test results. On the basis of the studies used for developing the search strategy, the proposed fields for abstraction include author last name, year, full journal name, reviewer’s initials, study type, article type, setting, geographic location (country and continent), and tools and method type (temporal and spatiotemporal). We will try to draw a distinction in the use of methods within the visual and analytic engines, if possible, especially related to machine learning, natural language processing, and other automated methods. In this regard, we have opted not to use the term *artificial intelligence* as it is nonspecific. Furthermore, the abstraction fields will include innovation and impact of the VA method/uptake of the method, target user/audiences, settings for the use of the VA solution, and potential application toward knowledge translation. Two reviewers will review and chart the data independently for each article.

### Results’ Synthesis and Presentation

Abstracted information from all the included articles will be synthesized, and the results will be presented to capture the extent of the literature. First, tables will provide the basic information on the types of studies included, the use of VA in various areas of population health or HSR, and the major tools and frameworks used. This overview will be followed by a narrative presentation of the synthesized mapping of the included literature. The tables and presentation will be developed considering the abstracted results. We are not limiting the review to being reported against a said framework at this point; however, we intend to use the guidance provided in Levy and Ellis’s paper on reporting reviews on information systems [63], which was selected on the basis of its subject-specific reporting. The authors cite the potential problems in reporting findings in such reviews, suggesting to place them in the wider context of the body of knowledge and the research itself, while building on a theoretical foundation [63].

## Results

As of late August 2019, the scoping review is in the full-text screening stage. Data synthesis will follow and the first results are expected to be submitted for publication in December 2019.

## Discussion

### Comparison With Prior Work

Recent scoping and systematic reviews on the related subjects of analytics and data mining show that VA is being increasingly taken up as a method of choice for big data in health care [1,6,19]. In population health and HSR, VA techniques are being applied to complex questions of service delivery and disease distribution [2,15,20]. Recent reviews include methods and approaches for evaluation of health visualizations and VA [24] as well as data mining applications and theoretical perspectives in health care analytics [22]. The proposed scoping review is novel in summarizing VA methods that have been either applied or proposed to use cases in population health and HSR, using structured or unstructured, complex big data from any or multiple source(s). To the best of our knowledge, there is no synthesis of literature in this area, which will add to the body of literature on these evolving methods of analysis toward complex health care data.

### Limitations

This scoping review methodology does not include book chapters, theses, short papers, editorials, nonpeer-reviewed reports, conference abstracts, and live websites using VA techniques for reasons mentioned above. We also limit the use of VA methods from 2005 onward that have been applied to population health and HSR. Finally, we do not explore subject-specific databases, such as from geography and computer science, which may limit our findings to proposed or established methods that have been either published or presented. However, we focus on casting a wide net to capture relevant methods for use cases in both population health and HSR. We devised the methodology in consultation involving a substantial number of multidisciplinary experts to advise on the rigor and feasibility of the review. We also hope to present the findings in 1 or more articles to illustrate the state of science for this important and emerging method.

### Conclusions

This scoping review will attempt to provide a foundational understanding of the current landscape of VA within population health and HSR. VA holds tremendous potential for contributing to the learning health systems approach, allowing complex data analysis, and visualization toward improving practices. Mapping the existing VA tools, strategies, and frameworks to health data will promote the use of these methods, which are being increasingly taken up for embedded research and future initiatives in health services. This scoping review protocol describes the design for the review on VA methods in population health and HSR, and it also lays out methodological challenges and steps taken toward ensuring rigor. The latter can be applied and developed by researchers beyond the subject area.

## Appendix

Multimedia Appendix 1 Medical Literature Analysis and Retrieval System Online search strategy.

## Abbreviations

EHR: electronic health record
EMR: electronic medical record
HSR: health services research
VA: visual analytics
