# Supplementary material for: Visual Analytic Tools and Techniques in Population Health and Health Services Research: Protocol for a Scoping Review
Source: JMIR Res Protoc. 2019 Oct 28;8(10):e14019. doi: 10.2196/14019 (PMC6913692; doi:10.2196/14019)
Supplement: Multimedia Appendix 1 [file resprot_v8i10e14019_app1.pdf]

Annex 1: Medline Search Strategy

Ovid MEDLINE(R) Epub Ahead of Print, In-Process & Other Non-Indexed Citations, Ovid MEDLINE(R) Daily and Ovid MEDLINE(R) **<1946 to February 26, 2019>**

#	Searches	Results
1	"visual* analytic*".tw,kf.	498
2	((health or healthcare) adj (analytic* or analy*)).ti,kf.	157
3	data visualization/	5
4	(visual* adj analy*).tw,kf.	4448
5	(information adj visual*).tw,kf.	446
6	(data adj visual*).tw,kf.	1616
7	(visual* adj platform?).tw,kf.	118
8	(visual* adj dashboard?).tw,kf.	20
9	(visual* adj representation?).tw,kf.	1711
10	(interactiv* adj3 (map* or graph*)).tw,kf.	1041
11	(visual* and (analy* or information* or data or platform? or dashboard? or representation?)).ti,kf.	11707
12	or/3-11	18488
13	Big data/	125
14	big data.tw,kf.	4900
15	((health or healthcare) adj3 data).tw,kf.	32400
16	exp Health services research/	156248

17	((health or healthcare) adj3 (research* or data or service? or deliver*)).tw,kf.	234548
18	exp Health Services Accessibility/	103235
19	Health Planning/	21374
20	exp Regional Health Planning/	38865
21	Health Resources/	11994
22	Healthcare Disparities/	13936
23	((health or healthcare) adj3 (equit* or equalit* or inequit* or inequalit* or disparit*)).tw,kf.	28701
24	((health or healthcare) adj3 (plan? or planning?)).tw,kf.	28398
25	((health or healthcare) adj3 evaluat*).tw,kf.	28202
26	((health or healthcare) adj3 (utiliz* or utilis* or access* or resourc*)).tw,kf.	68797
27	((health or healthcare) adj3 monitor*).tw,kf.	9584
28	Population Health/	374
29	exp Population Characteristics/	1823783
30	(population* adj3 health*).tw,kf.	40191
31	(population* adj3 (characteristic? or demographic?)).tw,kf.	29135
32	exp Population Surveillance/	64189
33	Public Health Systems Research/	18
34	surveillance.tw,kf.	159662
35	biosurveillance.tw,kf.	260
36	exp Public Health/	7247194
37	(public* adj3 health*).tw,kf.	249554
38	epidemiolog*.tw,kf.	381559

39	(administrative adj2 (data or database)).tw,kf.	11164
40	or/13-39	8019705
41	12 and 40	5689
42	1 or 2 or 41	6185
43	limit 42 to yr="2005 -Current"	4719
44	limit 43 to English language	4563

*All 12 seminal citations that are available in Medline are retrieved by the current search strategy.
